# Wasps' Nurseries

**Published:** 1893-08-26

**Authors:** 


### WASPS' NURSERIES.

The plague of wasps this year has been an additional trial to tempers already exasperated by the unusual heat. It certainly, therefore, seems a somewhat misplaced ambition on the part of the clergyman whom we hear has been so anxious to be the possessor of a wasp's nest superior in size to the one in the British Museum, that to the deep disgust of his neighbours he has been assiduously feeding the wasps in his garden with beer and sugar. It is hardly surprising that this ambition is not shared by the dwellers in the neighbouring houses.

